# Connective tissue diseases combined with chronic myelomonocytic leukemia and myelofibrosis: a case report and literature review

**DOI:** 10.3389/fimmu.2026.1685536

**Published:** 2026-05-14

**Authors:** Hai-Qin Yin, Sha Jin, Yao Fu, Hui-Ling Zhu, Xue-Fei Li

**Affiliations:** 1 Department of Rheumatology and Immunology, Jiujiang University Affiliated Hospital, Jiujiang, Jiangxi, China; 2 Department of Oncology, Jiujiang University Affiliated Hospital, Jiujiang, Jiangxi, China

**Keywords:** autoimmune diseases, azacytidine, chronic myelomonocytic leukemia, clonal hematopoiesis, connective tissue diseases, dual-targeting therapy, myelofibrosis, ruxolitinib

## Abstract

Connective tissue disease (CTD) is one of the common autoimmune diseases (AIDs). Chronic myelomonocytic leukemia (CMML) is a clonal hematopoietic stem cell disease characterized by peripheral blood monocytosis and bone marrow dysplasia, and it is frequently associated with autoimmune complications and secondary myelofibrosis (MF). This article reports a 52-year-old male patient with recurrent arthritis and fever as the main manifestations, for whom CTD was initially considered, and who received hormone and immunosuppressant combination therapy for 2 years 10 months. The patient still suffered from recurrent fever and severe anemia; further bone marrow cytology examinations revealed abnormal bone marrow hematopoiesis, and the final diagnosis was CTD complicated with CMML and MF (MF-3 grade). After the patient was transferred to the hematology department, we adopted the azacytidine + ruxolitinib dual-target regimen to simultaneously target autoimmune inflammation, malignant clonal hematopoiesis, and severe myelofibrosis. After treatment, the patient’s fever and other systemic symptoms were significantly relieved, monocytosis was corrected, and severe anemia and thrombocytopenia were markedly improved.

## Introduction

Autoimmune diseases (AIDs) are a class of diseases caused by the immune system mistakenly attacking the body’s own tissues or cells. Chronic myelomonocytic leukemia (CMML) is a myelodysplastic syndrome (MDS) and myeloproliferative neoplasm (MPN) characterized by cytopenias, persistent monocytosis (>1 × 10^9^/L), and morphological dysplasia, with a tendency to transform into acute myeloid leukemia (AML) (1). Chronic inflammation from AID promotes genomic instability and clonal hematopoiesis, while CMML-derived cytokines further drive autoimmunity. CMML is also frequently complicated by secondary myelofibrosis (MF), which exacerbates cytopenias and creates a triple comorbidity with connective tissue disease (CTD), which is diagnostically and therapeutically challenging.

The association between CMML and AID has been demonstrated, with approximately 30% of patients with CMML having or having had multiple systemic inflammations and AIDs (2). Overlapping symptoms include fever, cytopenia, and splenomegaly, leading to frequent misdiagnosis.

This article reports a rare case of CTD combined with CMML and severe MF (MF-3 grade). The purpose of this report was to clarify the clinical characteristics and diagnostic pitfalls of this triple comorbidity and to highlight the clinical value of the azacytidine + ruxolitinib dual-target regimen in the treatment of this complex condition so as to provide a reference for clinical diagnosis and treatment and reduce the rate of misdiagnosis and missed diagnosis.

## Case report

The patient was a 52-year-old man with no smoking history and no family history of rheumatic or malignant diseases. In July 2021, he presented with fever and ankle/knee pain, with body temperature ranging from 37.5 °C to 38.5 °C. Routine blood tests and monocyte counts showed normal results. Antinuclear antibody (ANA) (speckled pattern) was 1:320, Anti-Sjögren's syndrome antigen (SSA) antibody and Anti-Sjögren's syndrome B (SSB) antigen antibody were positive, IgG was 33.49 g/L, C-reactive protein (CRP) was 43.51 mg/L, Rheumatoid factor (RF) was 76.55 IU/mL, and anti-Cyclic Citrullinated Peptide (CCP) antibody and Anti-Neutrophil Cytoplasmic Antibody (ANCA) were negative. Labial gland biopsy showed normal findings, excluding Sjögren’s syndrome. MRI demonstrated inflammatory arthropathy without definite marrow lesions. After ruling out infection, endocrine disorders, and solid tumors, CTD was suspected. He was treated with methylprednisolone 20 mg qd, hydroxychloroquine 200 mg bid, and methotrexate 10 mg weekly. Symptoms resolved and inflammatory markers normalized within 4 weeks.

In May 2022, he developed recurrent fever and arthralgia. Laboratory tests revealed White Blood Cell (WBC) 12.93 × 10^9^/L, monocyte count 2.33 × 10^9^/L (18.0%), and CRP 40.14 mg/L. Infection was excluded; the patient refused bone marrow examination. Immunosuppressive therapy was adjusted: leflunomide 20 mg qd and tofacitinib 5 mg bid were added, with methylprednisolone tapered to 5 mg qd. Symptoms improved slightly, and low-dose steroid maintenance was continued.

In February 2023, he developed bacterial pneumonia and cutaneous herpes zoster, attributed to long-term immunosuppression. Tofacitinib and leflunomide were discontinued immediately. Anti-infective and antiviral treatment controlled infection within 2 weeks. Thereafter, recurrent irregular fever persisted. WBC was 9.76–12.30 × 10^9^/L, monocyte count was 1.78–2.16 × 10^9^/L (17.5–20%), and CRP was 36.49 mg/L. Chest and abdominal CT showed no abnormal findings. Peripheral blood smears showed 2% myelocytes and 1% metamyelocytes; bone marrow morphology and culture were normal. Cytogenetic and molecular tests were not performed, as clinical features favored infection and CTD flare rather than overt myeloid neoplasm. Methylprednisolone was increased to 20 mg qd, leading to the resolution of fever and normalized CRP within 2 weeks.

**Figure 1:**
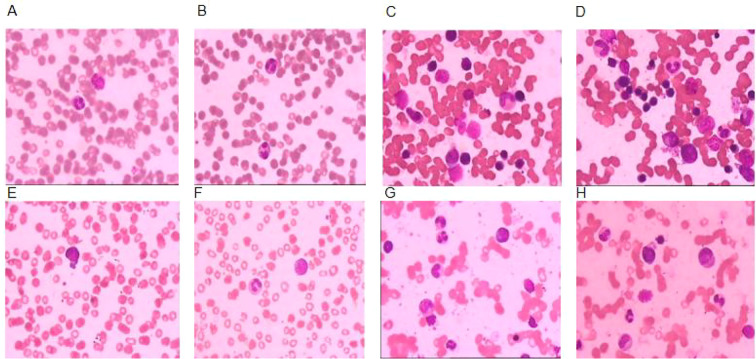
Morphological findings of peripheral blood and bone marrow in the patient. **(A, B)** Peripheral blood smear: 2% neutrophilic myelocytes and 1% neutrophilic metamyelocytes. **(C, D)** Bone marrow cytology: bone marrow nucleated cells showed hypercellularity, with increased and coarsely granulated cytoplasm in some granulocytic cells; erythroid hyperplasia was prominent without obvious dysplasia. **(E, F)** Peripheral blood smear: 1% blasts and 2% neutrophilic myelocytes. **(G, H)** Bone marrow cytology: bone marrow nucleated cells were hypercellular; some granulocytes showed increased and coarsely granulated cytoplasm; nucleated erythrocytes exhibited nuclear malformation. Nucleated erythrocytes and myelocytes were visible in the peripheral blood, and teardrop‐shaped erythrocytes were present in mature erythrocytes.

From February 2023 to May 2024 (15 months), he continued to have recurrent fever and arthralgia with fluctuating monocytosis and elevated CRP. Repeated imaging and laboratory tests showed no progressive abnormalities. Only symptomatic and supportive care was given, including intermittent low-dose glucocorticoids and Non-Steroidal Anti-Inflammatory Drugs (NSAIDs). No targeted immunosuppressants were restarted due to prior severe infectious complications. In May 2024, he was admitted with fatigue, persistent fever, severe diarrhea, myalgia, and shock due to severe dehydration. Laboratory results were as follows: WBC 9.47 × 10^9^/L, monocyte count 2.06 × 10^9^/L, Hemoglobin (Hb) 47 g/L, and Platelet (PLT) 67 × 10^9^/L. Workup for hemolytic anemia, ANCA, and antiplatelet antibodies yielded negative results; ANA was 1:100, and anti-SSA was positive. Abdominal ultrasound showed hepatosplenomegaly. Peripheral blood smears revealed 1% blasts and 2% myelocytes (**Figures 1E, F**). Bone marrow aspirate showed hypercellularity and erythroid dysplasia (**Figures 1G, H**). Flow cytometry detected 1.17% myeloblasts with abnormal differentiation, partial CD38 expression, and increased monocytes.

Peripheral blood MDS/MPN panel identified mutations in NRAS, CBL, ETNK1, PTPN11, SETBP1, ASXL1, and ZRSR2. BCR/ABL was negative. Karyotype analysis demonstrated 46,XY,t(3;12)(p12;q13) [30]. Bone marrow biopsy showed marked hypercellularity, 3% CD34+/CD117+ blasts, 10% CD68+ monocytes, lymphoid megakaryocytes, and severe interstitial fibrosis (MF-3 grade).

Based on the WHO 2022 diagnostic criteria and Cumulative Prognostic Scoring System – Molecular (CPSS-MOL) stratification, the patient was definitively diagnosed with high-risk chronic myelomonocytic leukemia-1 (CMML-1), secondary severe myelofibrosis (MF-3), and underlying CTD. Allogeneic Hematopoietic Stem Cell Transplantation (HSCT) was not feasible due to poor performance status (Eastern Cooperative Oncology Group (ECOG) 3), active systemic inflammation, lack of matched donor, and high transplant-related mortality risk in the setting of severe MF and prior severe infection. After excluding contraindications (no severe organ dysfunction, no active infection, and normal coagulation), the combination of azacitidine + ruxolitinib was initiated, targeting high-risk CMML-1, severe MF-3, and refractory CTD-associated inflammation. After four cycles, monocyte count decreased to 0.89 × 10^9^/L, Hb increased to 75 g/L, and PLT rose to 125 × 10^9^/L. Fever and arthralgia resolved; CRP normalized. No severe adverse events (myelosuppression, infection, and bleeding) occurred. The patient voluntarily discontinued treatment after cycle 4 for personal reasons (**Table 1**).

**Table 1:** Clinical manifestations, laboratory/imaging/pathology findings, treatment, and follow-up timeline.

Time period	Core clinical manifestations and symptoms	Key laboratory, imaging, and pathology findings (hematology → immunology/biochemistry → pathology → radiology)	Treatment	Follow-up (monitoring and outcome)
Jul 21	Fever (37.5 °C–38.5°C), ankle and knee pain	1. Hematology: normal complete blood count and monocyte count 2. Immunology: ANA 1:320 (speckled pattern), anti-SSA/SSB positive; IgG 33.49 g/L, CRP 43.51 mg/L, and RF 76.55 IU/mL; anti-CCP and ANCA negative 3. Pathology: normal labial gland biopsy 4. Radiology: MRI showed inflammatory arthropathy without bone marrow lesions	Methylprednisolone 20 mg qd, hydroxychloroquine 200 mg bid, and methotrexate 10 mg weekly	Symptoms resolved; inflammatory markers normalized within 4 weeks
May 22	Recurrent fever and arthralgia (ankle, knee, and foot)	1. Hematology: WBC 12.93 × 10^9^/L, monocyte count 2.33 × 10^9^/L (18.0%), and CRP 40.14 mg/L^2^. Others: infection and solid tumors excluded; patient refused bone marrow examination	Leflunomide 20 mg qd and tofacitinib 5 mg bid added; methylprednisolone tapered to 5 mg qd	Symptoms slightly improved; low-dose steroid maintenance continued
Feb 23	Bacterial pneumonia and cutaneous herpes zoster; persistent irregular fever	1. Hematology: WBC 9.76–12.30 × 10^9^/L, monocyte count 1.78–2.16 × 10^9^/L (17.5–20%), and CRP 36.49 mg/L; peripheral blood smear: 2% myelocytes and 1% metamyelocytes 2. Pathology: normal bone marrow morphology and microbial culture 3. Radiology: unremarkable chest and abdominal CT	Tofacitinib and leflunomide discontinued; anti-infective + antiviral therapy; methylprednisolone increased to 20 mg qd after infection control	Infection resolved in 2 weeks; fever and CRP normalized
February 2023 to May 2024	Recurrent fever and arthralgia (15 months)	1. Hematology: fluctuating monocytosis and elevated CRP 2. Others: no progressive abnormalities on repeated imaging and laboratory tests	Intermittent low-dose glucocorticoids and NSAIDs; no targeted immunosuppressants	Symptoms persisted; no further deterioration
May 24	Fatigue, persistent fever, severe diarrhea, myalgia, dehydration, and shock	1. Hematology: WBC 9.47 × 10^9^/L, monocyte count 2.06 × 10^9^/L, Hb 47 g/L, and PLT 67 × 10^9^/L; peripheral blood smear: 1% blasts and 2% neutrophilic myelocytes 2. Pathology: bone marrow aspirate: hypercellularity with erythroid dysplasia; flow cytometry: 1.17% abnormal myeloblasts; MDS/MPN mutations: NRAS, CBL, ETNK1, PTPN11, SETBP1, ASXL1, and ZRSR2; BCR/ABL negative; karyotype: 46,XY,t(3;12)(p12;q13) [30]; bone marrow biopsy: MF-3 grade 4. Radiology: abdominal ultrasound showed hepatosplenomegaly Diagnostic criteria: met WHO 2022 criteria for CMML-1 and MF-3	Azacitidine + ruxolitinib dual-target regimen	After 4 cycles: monocyte count normalized, Hb 75 g/L and PLT 125 × 10^9^/L; fever and arthralgia resolved; CRP normalized; no severe adverse events; patient discontinued treatment voluntarily

## Discussion

Autoimmunity is closely associated with a variety of hematological and non-hematological malignancies. The association between CMML and AIDs is not uncommon, and the incidence of AIDs (particularly vasculitis and CTD) among patients with bone marrow malignancies is relatively high. The clinical manifestations are diverse, mainly including systemic vasculitis, inflammatory arthritis, idiopathic thrombocytopenia, and idiopathic eosinophilia (3). Montoro J et al. found that 48% of patients with MDS/CMML were diagnosed with an AID, with ANA representing the most common abnormal serological immunological marker (23.2%) (4). Currently, there are few literature reports of the coexistence of CTD, CMML, and MF. Although this comorbidity phenomenon is rare, it is not accidental.

This case involves a patient with CTD complicated by CMML and severe MF (MF-3 grade)—a rare triple comorbidity that directly guided our therapeutic strategy. The coexistence of CMML and MF is the core rationale for combining ruxolitinib and azacitidine: ruxolitinib targets myelofibrosis-related myeloproliferative abnormalities, while azacitidine addresses CMML’s myelodysplastic and leukemic features (2, 5). A literature review confirmed the rarity of CMML concurrent with MF, with few reports of azacitidine–ruxolitinib combination for this dual condition, supporting the innovativeness of our approach in this triple comorbidity.

Briefly, CMML’s pathophysiology is driven by dysregulated myeloid hematopoiesis, with driver gene mutations and clonal hematopoiesis as key contributors, which, combined with MF-induced bone marrow fibrotic changes, exacerbated the patient’s hematological dysfunction (6, 7).

Cytogenetic and molecular abnormalities play key roles in predisposing to CMML–CTD–MF association and myelofibrosis development: CMML-related mutations (TET2, SRSF2, ASXL1, and RAS pathway genes) predispose to clonal hematopoiesis and autoimmune manifestations (7, 8), while JAK2 V617F, ASXL1, and SETBP1 mutations are linked to secondary myelofibrosis in CMML (2, 7, 9). Cytogenetically, abnormalities like t(3;12) may further drive disease progression (1). Our patient had NRAS, CBL, ETNK1, PTPN11, SETBP1, ASXL1, and ZRSR2 mutations and karyotype 46,XY,t(3;12)(p12;q13) [30], which, per the literature, likely explain the coexistence of CTD and severe MF (7, 8).

Infections are a common complication in this triple comorbidity: susceptibility stems from long-term immunosuppressive therapy for CTD and CMML-induced hematologic system disruptions, which impair immune surveillance and increase infection risk (10). Our patient’s prior bacterial lung infection and herpes zoster were linked to immunosuppressive therapy, consistent with this mechanism.

The overlapping symptoms of three diseases complicate diagnosis: both CTD and CMML present with fatigue, fever, and cytopenia (3, 4, 11), while CMML and MF are accompanied by splenomegaly and hepatomegaly, with possible elevated monocyte counts (2, 11, 12). However, splenic involvement may also occur in CTD, for example, in systemic lupus erythematosus and Felty’s syndrome. This overlap, including our patient’s hepatosplenomegaly, often leads to delayed diagnosis, highlighting the need to consider all three conditions in differential diagnosis.

CTD combined with CMML and MF is rare, and treatment should address all three conditions simultaneously. Most patients with CMML complicated by CTD have CMML-1. Corticosteroids are often effective, but approximately 40%–50% of cases require second-line therapy; relapse and steroid dependence are common after an initial response to steroids (13, 14). Hypomethylating agents (HMAs), the first-line therapy for CMML, including azacitidine and decitabine, are effective against CMML with an overall response rate of 40%–50% (2) and have been successfully used in about two-thirds of patients with AID manifestations (13). Currently, treatment options for MF are very limited, consisting mainly of improving clinical symptoms caused by splenomegaly and correcting anemia. With the use of JAK2 inhibitors, symptoms related to splenomegaly in MF can be improved (15), which also provides new therapeutic prospects for CMML (5, 16) and is widely used in various AIDs such as rheumatoid arthritis, systemic lupus erythematosus, psoriatic arthritis, and ankylosing spondylitis. Unfortunately, no effective treatment regimens have been reported for such CMML-MF patients. Sonja Heibl et al. suggested that the combination of JAK2 inhibitors and hypomethylating agents may be an attractive option for MPN patients with a CMML-like phenotype and for CMML patients with JAK2 mutations (17).

Based on the literature review above, the dual-targeting regimen of azacytidine + ruxolitinib was selected due to its ability to simultaneously target three pathological processes, and its rationality and superiority are reflected in the following three key aspects.

1. Dual targeting of the two core pathological processes: autoimmune inflammation and malignant clonal hematopoiesis.

Azacitidine, a hypomethylating agent, reverses aberrant DNA methylation in CMML clones, suppresses abnormal clonal proliferation, and corrects persistent monocytosis. Ruxolitinib, a selective JAK1/JAK2 inhibitor, blocks the JAK–STAT pathway, a key hub linking immune inflammation and clonal expansion in CTD–CMML. It inhibits pro-inflammatory cytokines (TNF-α, IL-6, and IFN-γ) to alleviate CTD-related systemic inflammation and also restrains abnormal myeloproliferation in CMML (2, 5). Together, they achieve simultaneous targeted control of both autoimmune inflammation and malignant clonal hematopoiesis.

2. Synergistic efficacy against myelofibrosis and hematopoietic failure.

The patient had grade 3 MF, a severe CMML complication that worsens bone marrow failure, anemia, thrombocytopenia, and hepatosplenomegaly. Ruxolitinib, first-line for MF, reduces pro-fibrotic cytokines (e.g., TGF-β), relieves marrow fibrosis, and improves cytopenias and splenomegaly. It also attenuates the inflammatory marrow microenvironment and enhances the anti-clonal effect of azacitidine (5, 18, 19). Azacitidine mitigates myelodysplasia, helps restore normal hematopoiesis, and lowers pro-inflammatory cytokine secretion by abnormal monocytes. The combination of azacytidine and ruxolitinib has potential synergy for reducing spleen length and improving bone marrow (BM) fibrosis (20).

3. Good safety and clinical feasibility, avoiding the limitations of conventional immunosuppressive therapy.

The patient had a history of severe infectious complications (bacterial lung infection and herpes zoster) induced by long-term conventional immunosuppressive therapy (tofacitinib + leflunomide + glucocorticoids), which made reinitiation of high-dose immunosuppressants risky. Azacitidine–ruxolitinib acts via targeted inhibition rather than non-specific immunosuppression, preserving normal immunity. A phase I/II trial (n = 50) supported the safety of ruxolitinib in CMML (5, 21). The preliminary data of combining ruxolitinib and azacytidine in MDS/MPN overlap syndromes (NCT01787487) indicated a good tolerance of the combination and a response rate of 57% in patients with MDS/MPNs, of which 17/35 (48%) had CMML (22). No severe adverse events occurred during four cycles, with marked improvements in inflammation and hematologic parameters, supporting good safety and feasibility in this high-risk population. This requires clinical trials with larger sample sizes to confirm.

## Conclusion

CTD complicated by CMML and severe MF is a rare, diagnostically challenging triple comorbidity, with overlapping symptoms often leading to delayed recognition. Cytogenetic and molecular abnormalities are key drivers of this association, explaining the coexistence of autoimmune and fibrotic features. The dual-target therapeutic approach effectively addresses the complex pathological processes of this comorbidity, offering a safe and feasible option for patients in whom conventional immunosuppression is risky. This case highlights the importance of considering all three conditions in differential diagnosis and individualizing treatment based on comorbidities. Multidisciplinary collaboration between rheumatologists and hematologists is crucial to reduce misdiagnosis and select optimal therapies. Further multicenter, large-sample studies are needed to validate the long-term efficacy of this dual-target regimen in similar patients.

## Data Availability

The original contributions presented in the study are included in the article/supplementary material. Further inquiries can be directed to the corresponding author.
